# Network toxicology reveals glyphosate mechanisms in kidney injury and cancer​​

**DOI:** 10.1038/s41598-025-17067-1

**Published:** 2025-08-24

**Authors:** Yiling Dong, Jufan Zhu

**Affiliations:** 1 General Practice Department, Tongxiang Wutong Street Community Health Service Center, Jiaxing, 314500 Zhejiang China; 2 Institute of Organoid Technology, Kunming Medical University, Kunming, 650500 Yunnan China

**Keywords:** Agrochemical, Kidney cancer, Kidney injury, Network toxicology, Molecular docking, Computational biology and bioinformatics, Diseases

## Abstract

Molecular mechanisms underlying glyphosate-induced nephrotoxicity and carcinogenicity were investigated through integrated network toxicology, molecular docking, and dynamics simulations. Screening identified 47 potential glyphosate targets; intersection analysis yielded 20 kidney injury and 31 kidney cancer shared targets. Protein-protein interaction networks highlighted matrix metalloproteinases (MMP9, MMP2, MMP8, MMP3) and PLG as topological hubs. Pathway enrichment revealed significant alterations in extracellular matrix reorganization and nitrogen metabolism. Molecular modeling demonstrated stable glyphosate binding within catalytic domains of MMPs (affinities: −5.03 to − 6.29 kcal/mol), with dynamics simulations confirming persistent complex formation over 100 ns. Results indicate MMP-mediated dysregulation of structural homeostasis, alongside metabolic pathway perturbation, as contributory factors in glyphosate-associated renal pathology. The prominence of MMPs across target networks and functional analyses suggests their role as molecular conduits for glyphosate toxicity.

## Introduction

Glyphosate, a broad-spectrum systemic herbicide, has become a cornerstone in modern agriculture since its introduction in the 1970s^1^. Its global prevalence can be attributed to its high efficacy, low cost, and persistence in the environment, making it the most extensively used herbicide worldwide^2^. Glyphosate inhibits the enzyme 5-enolpyruvylshikimate-3-phosphate (EPSP) synthase, a key component of the shikimate pathway critical for plant growth^3^. While animals lack this pathway, leading initially to assumptions about glyphosate’s limited toxicity to humans and other mammals, increasing evidence of environmental persistence and widespread exposure has prompted concerns about its health impacts^4^. Glyphosate and its main metabolite, aminomethylphosphonic acid (AMPA), have been detected in water, soil, and food, highlighting the extent of human exposure^5^. These findings have intensified a global debate regarding glyphosate’s safety, underscoring the need for thorough toxicological studies to evaluate its potential health risks.

Mounting evidence has intensified concerns about the nephrotoxic and carcinogenic potential of glyphosate. Studies indicate that chronic glyphosate exposure can trigger oxidative stress, inflammation, and apoptosis in renal cells, culminating in structural and functional kidney damage^6–8^. Epidemiological data further suggest a link between glyphosate exposure and higher rates of renal diseases, including chronic kidney disease and renal cell carcinoma^9^. Although the underlying mechanisms are not fully understood, these findings underscore glyphosate’s possible role in renal toxicity and carcinogenesis. Both in vitro and in vivo studies reveal glyphosate’s capacity to disrupt mitochondrial function, modulate gene expression involved in detoxification, and generate reactive oxygen species (ROS), collectively contributing to renal cell injury^10,11^. Given these findings, there is an urgent need to unravel the molecular pathways by which glyphosate may drive kidney injury and cancer progression, especially under conditions of prolonged exposure.

Network toxicology offers a transformative framework for dissecting the complex biological interactions triggered by chemical exposures such as glyphosate. Unlike traditional toxicology, which often focuses on individual molecular pathways or isolated targets, network toxicology employs systems biology to analyze the chemical’s impact across interconnected molecular networks^12^. By constructing comprehensive networks of potential targets, signaling pathways, and biological processes, this approach provides a multi-level perspective on glyphosate’s cellular and systemic effects. Network toxicology thus enables the identification of critical nodes—hub proteins and key signaling cascades—that may drive glyphosate’s toxic impact on kidney injury and cancer, uncovering broader toxicity patterns that conventional approaches might overlook^13^. This method therefore enhances our understanding of glyphosate’s complex toxicological profile and its underlying molecular mechanisms.

Computational approaches play a vital role within the framework of network toxicology by enabling the prediction and prioritization of potential interactions between small molecules like glyphosate and complex biological systems. Specifically, methodologies such as molecular docking and simulation analysis are employed to predict the binding affinity and favored orientation of glyphosate with proteins implicated in critical cellular processes—particularly those related to renal function and carcinogenesis (e.g., key enzymes, receptors, or transporters identified from network analyses^14,15^. This step is crucial as it helps delineate the molecular initiating events—the very first interactions at the protein level—that could set in motion the downstream cascades ultimately manifesting as nephrotoxicity or carcinogenicity. Thus, integrating computational prediction of these interactions within the broader network toxicology paradigm provides a powerful strategy for identifying plausible mechanistic pathways and focal points for further experimental investigation of glyphosate’s toxic effects^16,17^.

This study aims to utilize network toxicology, molecular docking and molecular dynamics simulation to systematically investigate the molecular targets and pathways implicated in glyphosate-induced renal injury and carcinogenesis. By combining computational analyses with toxicological data, we seek to identify key biological pathways affected by glyphosate exposure and clarify the molecular mechanisms underlying its nephrotoxic and carcinogenic effects. This work aims to offer a comprehensive assessment of glyphosate’s impact on renal health, providing critical insights to inform regulatory guidelines and public health policies regarding its use in agriculture. Through this research, we strive to deepen understanding of glyphosate’s toxicological profile and support the development of preventive strategies against glyphosate-associated renal diseases.

### Methods

### Network toxicological analysis

For toxicological analysis of glyphosate, two databases, ADEMTlab3.0^18^ and admetSAR3.0^18^, were utilized. The SMILES sequence of glyphosate was first obtained from the PubChem database^19^. This SMILES notation was then input into each database, and the predictive results were subsequently downloaded for further analysis.

### Acquisition of targets of glyphosate

The SMILES structure of glyphosate was obtained from the PubChem database. Subsequently, the SDF file of glyphosate was uploaded to the PharmMapper^20^ and SwissTargetPrediction^21^ databases to predict potential targets. The prediction results from both platforms were then merged, and duplicate targets were removed, resulting in a refined target library for glyphosate.

### Acquisition of disease-related targets

Target genes associated with kidney cancer and kidney injury were downloaded from databases including Genecards^22^ and OMIM^23^. The predicted targets from these databases were then combined, with duplicates removed, resulting in a curated disease target library.

### The intersection of drug targets and disease targets

The intersection of drug and disease targets was primarily conducted using the Venn website^24^. Briefly, the glyphosate target library and the target library for cancer or kidney injury were uploaded to the relevant section of the website and submitted to obtain the intersection results.

### Protein-protein interaction (PPI) network

The protein-protein interaction (PPI) network was constructed based on the intersection of identified targets using the STRING database^25^with the species parameter set to “Homo sapiens” and a minimum interaction confidence score of 0.4. The network visualization was generated in Cytoscape 3.10.2^26^. Additionally, the top 10 targets were ranked by degree values calculated via the cytoHubba plugin in Cytoscape 3.10.2.

### Gene ontology (GO) and kyoto encyclopedia of genes and genomes (KEGG) pathway enrichment analyses

Gene Ontology (GO)^27^ and Kyoto Encyclopedia of Genes and Genomes (KEGG)^28–31^ enrichment analyses for intersecting targets were performed using the DAVID database^32^ and Bioinformatics platfor^33^applying a significance threshold of (*P* < 0.05).

### Drug-target-pathway network building

The drug-target-pathway network provides an overview of how the drug modulates specific pathways through its key targets, highlighting the connections between critical targets and the drug to identify the most essential targets and pathways. Initially, the TSV file generated from previous analyses was uploaded to Cytoscape 3.10.2, followed by the drug-target mapping table. The two files were then merged, and the resulting property table was imported for further analysis^34^.

### Molecular docking

Target protein structures were obtained from the PDB database (http://www.rcsb.org/) and saved in PDB format, while glyphosate was saved in Mol2 format. The target proteins were subsequently prepared by hydrogenation and dehydration, and the ligand was optimized using PyMOL^35^. Molecular docking and conformational scoring were then performed using AutoDock Vina^35^.

### Molecular dynamics simulation

**Fig. 1:**
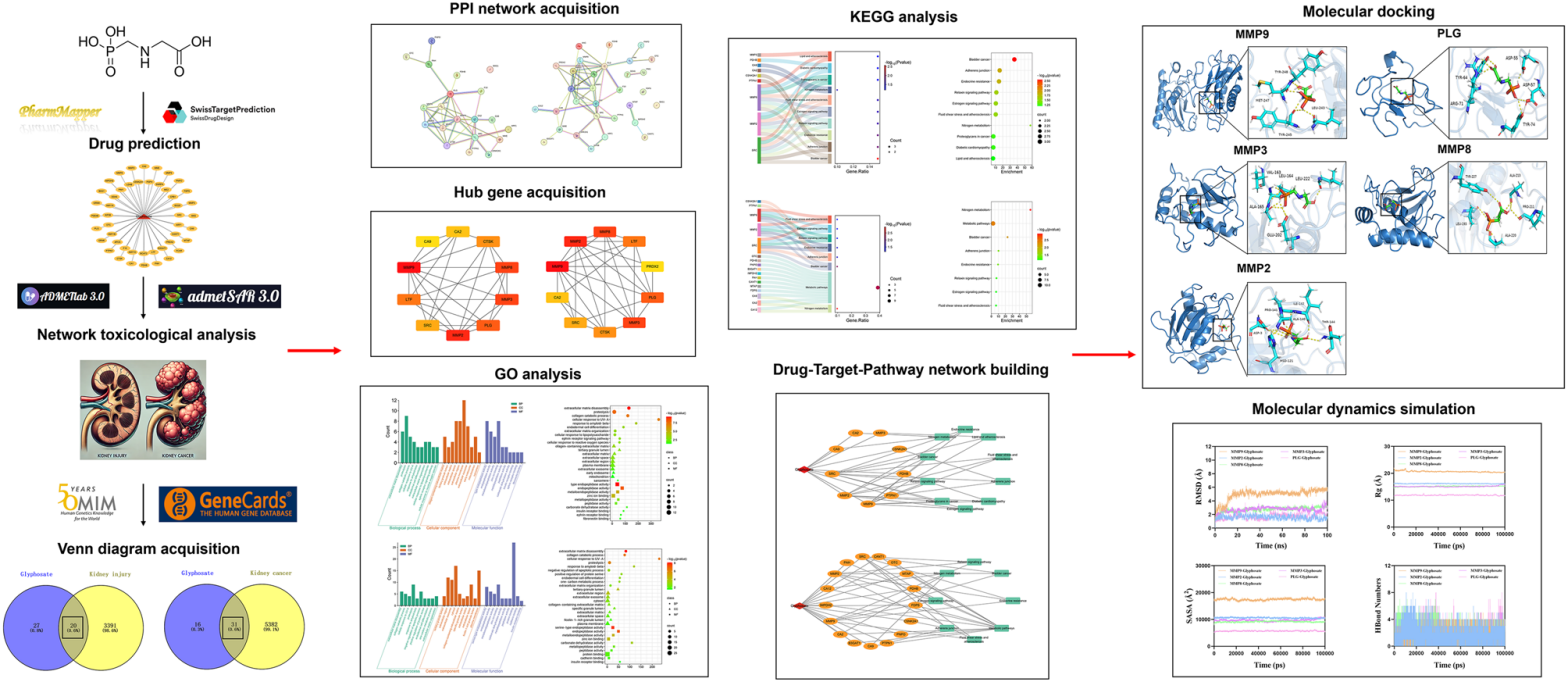
Integrative workflow for the study.

Molecular dynamics simulations were performed using GROMACS 2022 with the following protocol: The protein structure was parameterized using the CHARMM36 force field through GROMACS’s pdb2gmx tool, while ligand parameters were obtained from the CGenFF force field via the AutoFF web server^36^. The system was solvated in a cubic TIP3P water box extending 1 nm from the protein surface, and neutralized by adding appropriate ions using the gmx genion tool. Electrostatic interactions were calculated using the Particle Mesh Ewald (PME) method with a 1.0 nm cutoff distance, and all bonds were constrained using the SHAKE algorithm with a 1 fs integration time step implemented through the Verlet leapfrog algorithm. Prior to production runs, the system underwent energy minimization consisting of 3000 steps of steepest descent followed by 2000 steps of conjugate gradient optimization, performed sequentially with (1) solute constraints for water minimization, (2) counterion constraints, and (3) no restraints for full system minimization. Production simulations were carried out under NPT ensemble conditions at 310 K for 100 ns, during which structural properties including root-mean-square deviation (RMSD), root-mean-square fluctuation (RMSF), hydrogen bonds (HBonds), radius of gyration (Rg), and solvent accessible surface area (SASA) were calculated using GROMACS analysis tools (gmx rms, gmx rmsf, gmx hbond, gmx gyrate, and gmx sasa, respectively), following established protocols for protein-ligand interaction analysis^37–40^. The flow chart outlining the study is presented in Fig. 1 below.

## Results

### Prediction of targets in glyphosate

**Fig. 2:**
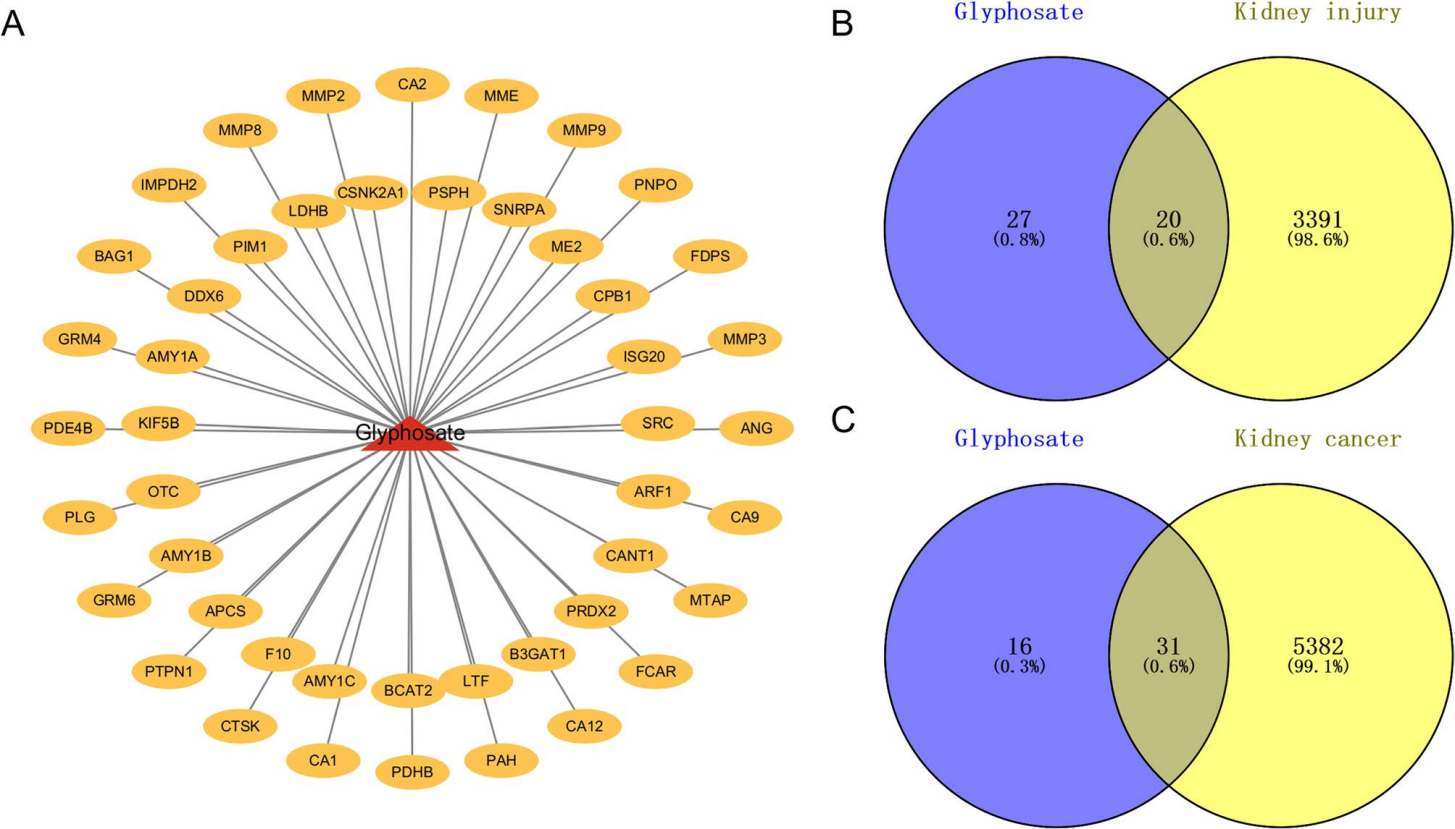
Intersection of targets between glyphosate-induced kidney injury and kidney cancer. (**A**) Compound-target network of glyphosate; (**B**) Venn diagram of potential targets associated with glyphosate-induced kidney injury; (**C**) Venn diagram of potential targets associated with glyphosate-induced kidney cancer.

Data on the identified potential drug targets, comprising 47 entries, were obtained from the PharmMapper and SwissTargetPrediction databases and subsequently visualized using Cytoscape 3.10.2 (Fig. 2A).

### Toxicity analysis of glyphosate

Predictions from the ADMETlab3.0 and admetSAR3.0 databases indicated that glyphosate’s co-toxicity primarily involves kidney injury (Table 1). Additionally, the databases predicted carcinogenicity, neurotoxicity, respiratory toxicity, and ototoxicity for glyphosate, although these effects were reported by only one database. Given its carcinogenic potential, the molecular mechanisms underlying glyphosate-induced kidney injury and kidney cancer will be further investigated.

### Potential targets of glyphosate-induced kidney injury and kidney cancer

A total of 3,411 potential targets for kidney injury and 5,413 for kidney cancer were identified using the GeneCards and OMIM databases.Venn diagram analysis revealed 20 intersecting targets between glyphosate and kidney injury, and 31 intersecting targets between glyphosate and kidney cancer. (Figure 2B C).

### PPI network and hub targets identification

**Fig. 3:**
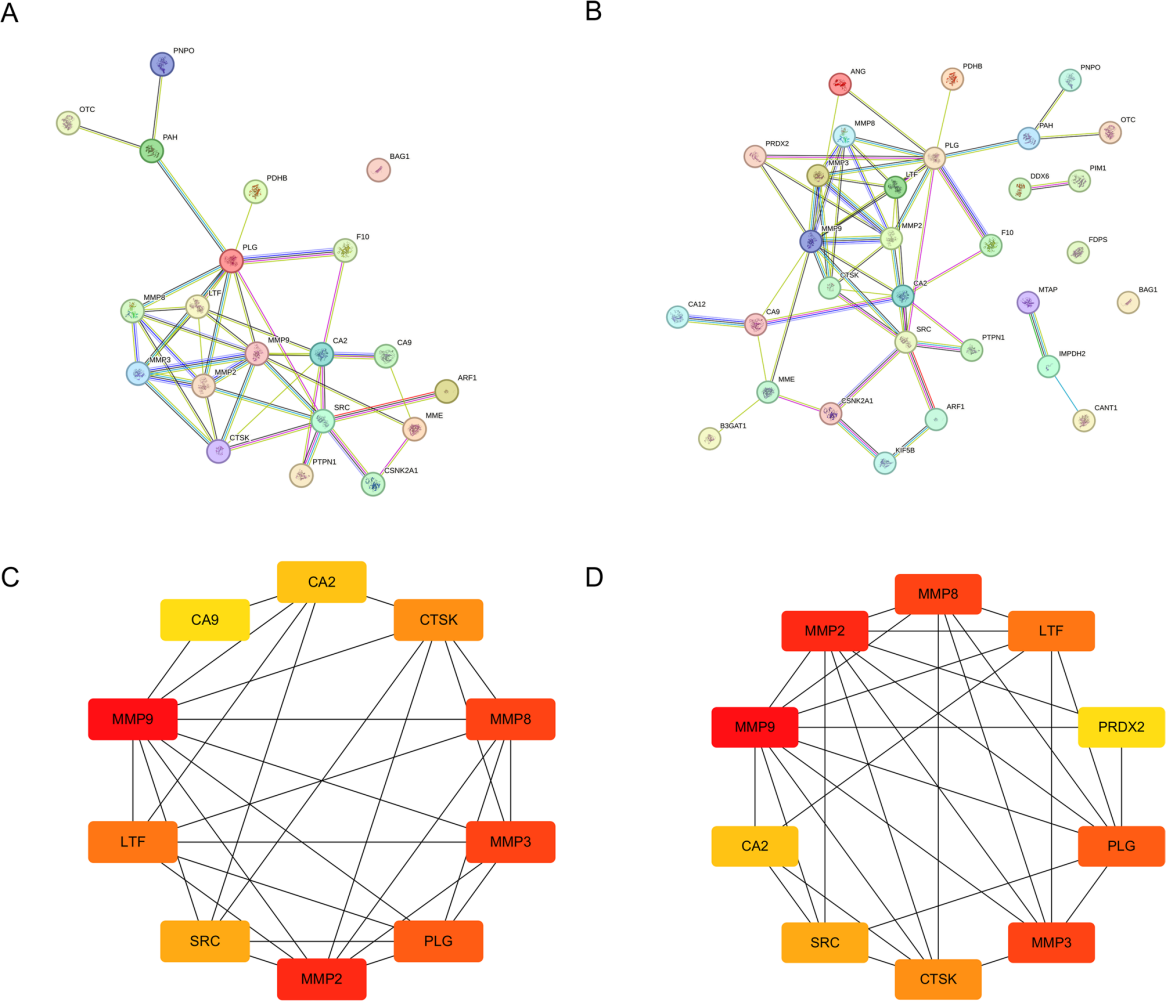
Target protein interaction network (PPI). PPI network diagram. (**A**) PPI network diagram of glyphosate-induced kidney injury; (**B**) PPI network diagram of glyphosate-induced kidney cancer; (**C**) The top10 targets were visualized in Cytoscape 3.10.2. of glyphosate-induced kidney injury; (**D**) The top10 targets were visualized in Cytoscape 3.10.2. of glyphosate-induced kidney cancer.

To elucidate the protein-protein interaction (PPI) networks involved in glyphosate-induced kidney injury and kidney cancer, intersecting targets were imported into the STRING database. In these networks, nodes represent proteins, while edges signify protein-protein interactions. Figure 3 A depicts the PPI network associated with glyphosate-induced kidney injury, whereas Fig. 3B shows the network related to glyphosate-induced kidney cancer. Subsequently, targets were ranked by degree values, with the top 10 targets visualized using Cytoscape 3.10.2.

The analysis identified 10 hub targets for glyphosate-induced kidney injury: MMP9, MMP2, MMP8, MMP3, PLG, LTF, CTSK, SRC, CA2, and CA9. Similarly, 10 hub targets were identified for kidney cancer: MMP9, MMP2, MMP8, MMP3, PLG, LTF, CTSK, SRC, CA2, and PRDX2 (Figs. 3 C and 3D). In these visualizations, node color intensity correlates with degree value, with a redder hue indicating a higher degree.

### GO enrichment analyses

**Fig. 4:**
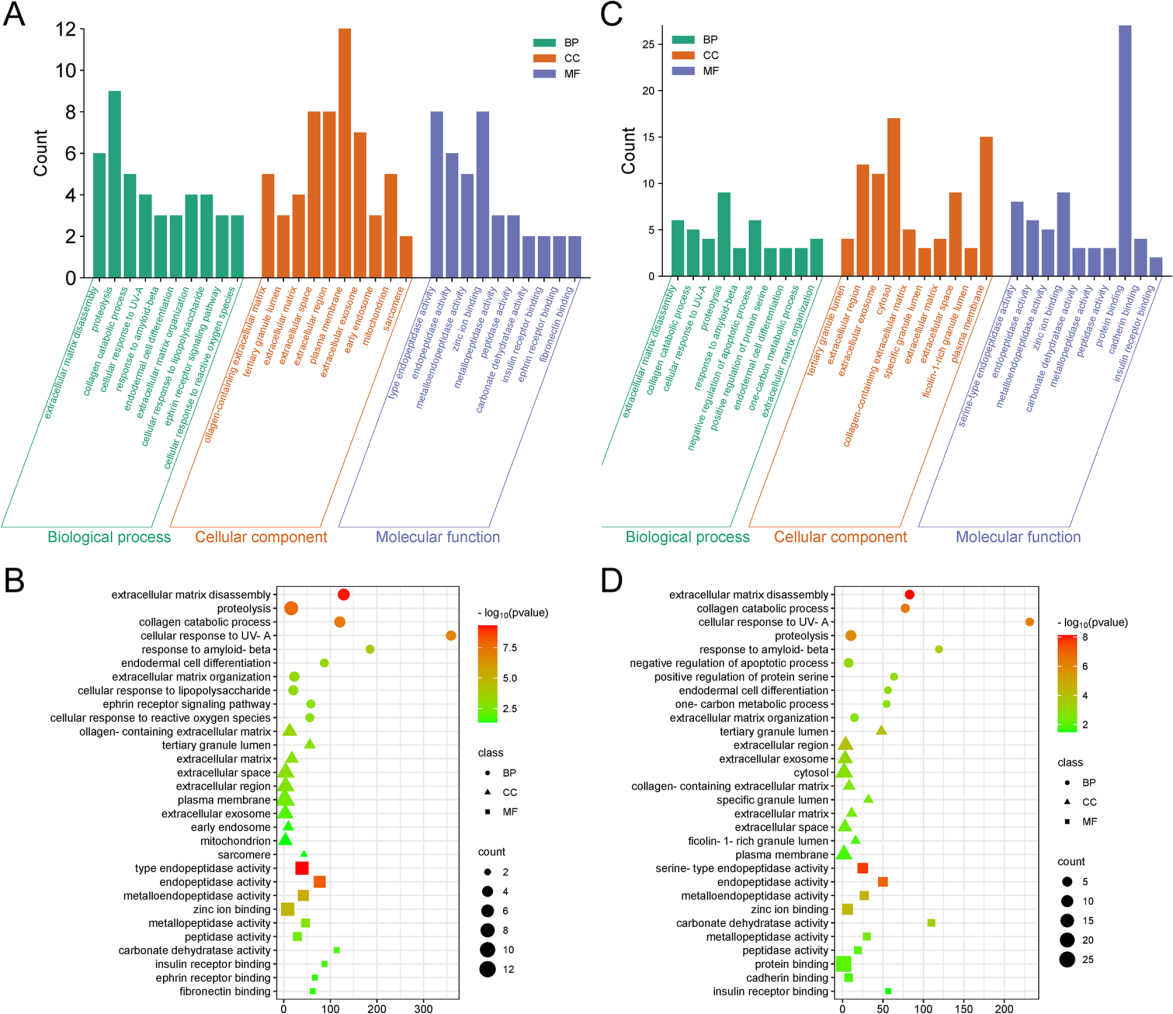
GO analysis. (**A**,** B**) GO diagram of glyphosate-induced kidney injury; (**C**,** D**) GO diagram of glyphosate-induced kidney cancer.

The intersecting targets associated with glyphosate-induced kidney injury and kidney cancer were analyzed for Gene Ontology (GO) enrichment using the DAVID database, covering biological processes (BP), cellular components (CC), and molecular functions (MF). As shown in Fig. 4A, the GO analysis for glyphosate-induced kidney injury revealed prominent biological processes, including proteolysis, extracellular matrix disassembly, and collagen catabolic process. The main cellular components identified were the plasma membrane, extracellular space, and extracellular region, while key molecular functions included endopeptidase activity, zinc ion binding, and endopeptidase activity. Figure 4B highlights GO features with a particular focus on p-values, showing that endopeptidase activity exhibited the largest p-value, whereas sarcomere displayed the smallest p-value.

Additionally, GO analysis for glyphosate-induced kidney cancer was performed using the DAVID database. The key biological processes identified included proteolysis, negative regulation of apoptosis, and extracellular matrix disassembly. Cellular components were primarily associated with the cytosol, plasma membrane, and extracellular region, while molecular functions involved protein binding, zinc ion binding, and serine-type endopeptidase activity (Fig. 4C). As shown in Fig. 4D, which visualizes p-values for glyphosate-induced kidney cancer, extracellular matrix disassembly displayed the highest p-value, whereas insulin receptor binding had the lowest.

### KEGG pathway enrichment analyses

**Fig. 5:**
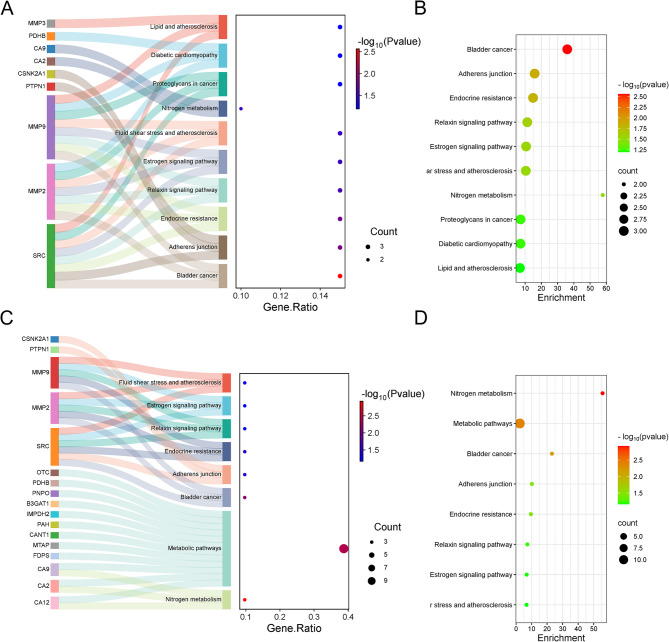
KEGG pathway analysis. (**A**,** B**) KEGG pathway diagram of glyphosate-induced kidney injury. (**C**,** D**) KEGG pathway diagram of glyphosate-induced kidney cancer.

Significant signaling pathways related to the intersecting targets were identified through KEGG pathway analysis. The top 10 KEGG pathways associated with glyphosate-induced kidney injury, ranked by p-value, are presented in Fig. 5A ,B, while the top 8 pathways for glyphosate-induced kidney cancer are shown in Fig. 5C,D. Pathways involved in glyphosate-induced kidney injury include bladder cancer, adherens junction, endocrine resistance, relaxin signaling pathway, estrogen signaling pathway, fluid shear stress and atherosclerosis, nitrogen metabolism, proteoglycans in cancer, diabetic cardiomyopathy, and lipid and atherosclerosis. The pathways related to glyphosate-induced kidney cancer include nitrogen metabolism, metabolic pathways, bladder cancer, adherens junction, endocrine resistance, relaxin signaling pathway, estrogen signaling pathway, and fluid shear stress and atherosclerosis.

As shown in Fig. 5A ,B, within the pathways associated with glyphosate-induced kidney injury, the bladder cancer pathway exhibits the lowest p-value, highlighting genes such as MMP9, MMP2, and SRC. In Fig. 5C, D, the nitrogen metabolism pathway displays the lowest p-value for glyphosate-induced kidney cancer, featuring genes such as CA9, CA2, and CA12. Notably, nitrogen metabolism emerges as the pathway with the highest enrichment in both glyphosate-induced kidney injury and kidney cancer.

### Glyphosate -induced kidney injury and kidney cancer network analysis

**Fig. 6:**
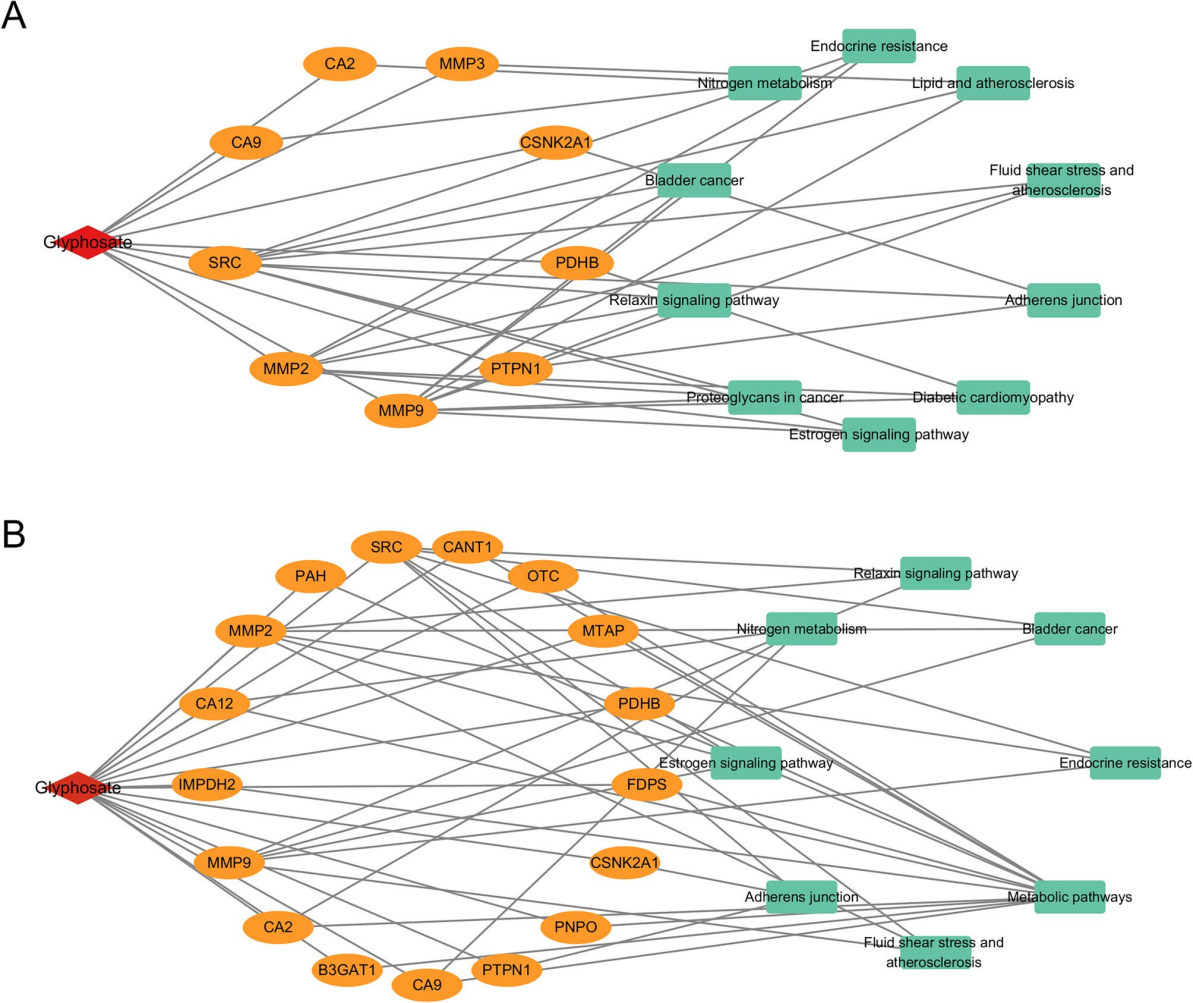
Glyphosate-induced kidney injury and kidney cancer network analysis. (**A**) “Drug-target-pathway” network diagram illustrating glyphosate-induced kidney injury; (B) “Drug-target-pathway” network diagram illustrating glyphosate-induced kidney cancer.

A “drug-target-pathway” network diagram illustrating glyphosate-induced kidney injury and kidney cancer was constructed using Cytoscape, as shown in Fig. 6. In this diagram, red nodes represent glyphosate, orange nodes denote hub targets, and green nodes signify pathways. The network provides a comprehensive view of the hub targets, associated pathways, and the degree of glyphosate-induced effects on kidney injury and kidney cancer.

### Molecular docking analysis

**Fig. 7:**
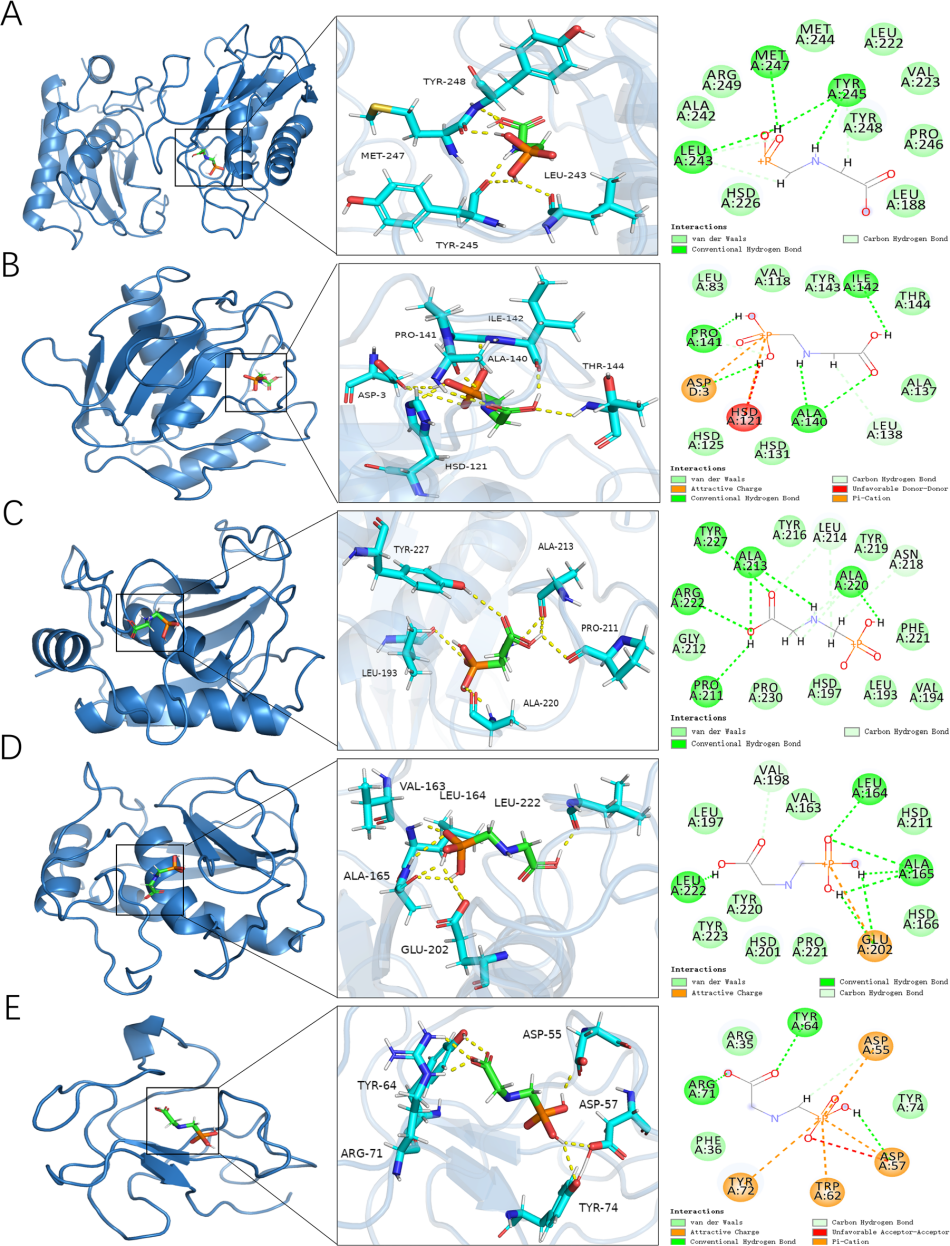
Three-dimensional and two-dimensional docking patterns and interactions of glyphosate with the hub targets. (**A**) MMP9; (**B**) MMP2; (**C**) MMP8; (**D**) MMP3; (**E**) PLG.

Molecular docking revealed specific binding interactions between glyphosate and key protein targets. As presented in Table 2, glyphosate demonstrates specific binding affinities with distinct amino acid residues. For MMP9, van der Waals interactions were observed with residues Arg249, Ala242, His226, Leu188, Pro246, Tyr248, Val223, Leu222, and Met244, while hydrogen bonds formed with Met247, Tyr245, and Leu243. The calculated binding affinity for the MMP9-glyphosate complex was − 5.93 kcal/mol. Glyphosate interacted with MMP2 via van der Waals forces at Tyr143, Val118, Leu83, His125, His131, Ala137, and Thr144. An unfavorable donor-donor clash occurred with His121, hydrogen bonds formed with Ile142, Pro141, Ala140, and Leu138, an attractive charge interaction with Asp3, and a pi-cation interaction with His121. The MMP2-glyphosate binding affinity was − 5.79 kcal/mol. MMP8 engaged glyphosate through van der Waals interactions at Tyr216, Tyr219, Phe221, Val194, Leu193, His197, Pro230, and Gly212, and hydrogen bonds with Leu214, Asn218, Ala220, Ala213, Tyr227, Arg222, and Pro211, yielding a binding energy of − 6.29 kcal/mol. Interactions with MMP3 involved van der Waals contacts at Val163, Leu197, Tyr220, Tyr223, His201, Pro221, His116, and His211, hydrogen bonds with Leu164, Ala165, Leu222, and Val198, and an attractive charge interaction with Glu202, resulting in a binding affinity of − 5.43 kcal/mol. Finally, PLG demonstrated van der Waals interactions with Tyr74, Arg35, and Phe36; hydrogen bonds with Tyr64, Arg71, Asp57, and Asp55 (alongside an unfavorable acceptor-acceptor clash with Asp57); attractive charge interactions with Tyr72 and Trp62; and pi-cation interactions with Asp57 and Asp55. The PLG-glyphosate complex had a binding affinity of − 5.03 kcal/mol​ (Fig. 7).

**Table 1:** The prediction toxicity of Glyphosate.

Property	Database	Probability
Drug-induced Nephrotoxicity	ADMETlab 3.0	0.999
Nephrotoxicity	admetSAR3.0	1.000
Carcinogenicity	ADMETlab 3.0	0.896
Drug-induced Neurotoxicity	ADMETlab 3.0	0.646
Respiratory toxicity	admetSAR3.0	1.000
Ototoxicity	ADMETlab 3.0	0.448

**Table 2:** Molecular docking results of glyphosate interactions with key protein targets.

Receptor	Binding energy (Kcal/mol)
MMP9	-5.93
MMP2	-5.79
MMP8	-6.29
MMP3	-5.43
PLG	-5.03

### Molecular dynamics simulation analysis

**Fig. 8:**
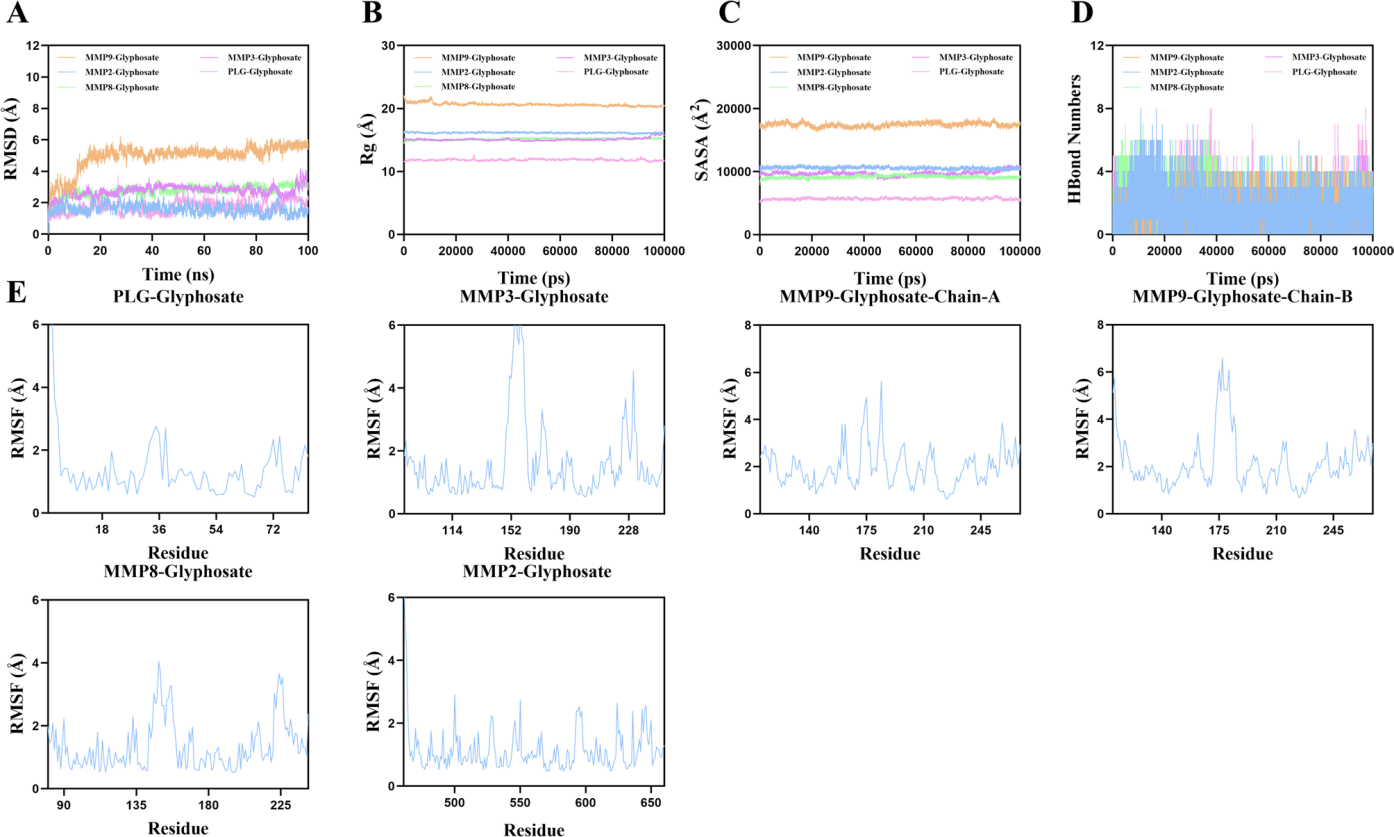
Molecular dynamics simulation of protein-ligand complexes.​ (**A**) RMSD of the protein-ligand complex over simulation time; (**B**) Rg of the protein-ligand complex over time; (**C**) SASA of the protein-ligand complex over time; (**D**) HBonds in the protein-ligand complex over time; (**E**) RMSF of residue backbone atoms in the protein-ligand complex.​.

The molecular dynamics simulation results demonstrate that root-mean-square deviation (RMSD) serves as an excellent indicator for evaluating the conformational stability of protein-ligand complexes, with lower values indicating better stability. As shown in Fig. 8A, the MMP3-Glyphosate complex exhibited stable fluctuations between 20 and 85 ns before showing a slight upward trend while remaining below 2.7Å, similar to other complexes which reached equilibrium at different timepoints (MMP8 at 10 ns ~ 3Å, MMP2 at 20 ns ~ 1.5Å, MMP9 at 80 ns ~ 5.5Å, and PLG at 90 ns ~ 2.3Å). The radius of gyration (Rg) analysis revealed minimal structural expansion, with all complexes achieving equilibrium around 2.3Å after 90 ns (Fig. 8B), suggesting stable compactness without significant conformational changes. Solvent accessible surface area (SASA) measurements showed slight fluctuations (Fig. 8C), indicating modest alterations in the binding microenvironment upon ligand interaction. Hydrogen bond analysis demonstrated robust interactions, with MMP3-Glyphosate maintaining ~ 3 bonds (range 0–6), MMP8 ~ 4 bonds (0–7), MMP2 ~ 4 bonds (0–8), and MMP9 ~ 4 bonds (0–5) (Fig. 8D). The root-mean-square fluctuation (RMSF) values remained relatively low (0.7–2.1Å) (Fig. 8E), confirming restricted residue flexibility and high stability. These comprehensive analyses align with findings from multiple studies^37–40^where stable RMSD trajectories (typically < 3Å over 100ns simulations), consistent Rg patterns, and sustained hydrogen bonding (often 2–5 bonds) were observed in validated protein-drug complexes. The collective data confirms that Glyphosate forms stable complexes with MMP3, MMP8, MMP2, MMP9 and PLG targets through favorable binding interactions and structural compatibility.

## Discussion

This study provides a comprehensive investigation into the molecular mechanisms by which glyphosate may contribute to kidney injury and kidney cancer, employing an array of bioinformatics tools for target prediction, toxicity assessment, pathway enrichment analysis, molecular docking and molecular dynamics simulation. By integrating data from PharmMapper and SwissTargetPrediction, 47 potential glyphosate targets were identified, establishing a robust foundation for further toxicity and mechanistic exploration. The toxicity profiles predicted by ADMETlab3.0 and admetSAR3.0 highlight kidney injury as a primary risk associated with glyphosate, alongside additional concerns of carcinogenicity, neurotoxicity, respiratory toxicity, and ototoxicity. The indicated potential for carcinogenicity, although corroborated by only a single database, underscores the necessity of investigating glyphosate’s long-term impacts on renal health and cancer development.

Traditional toxicological approaches, such as animal and cell-based assays, continue to serve as the benchmark for assessing the toxicity of glyphosate. These methods, which encompass acute toxicity studies in mice and extended testing in rats, aim to ensure safety prior to human exposure. Despite their effectiveness in reflecting in vivo and in vitro toxic effects, these techniques are both costly and ethically contentious due to their reliance on animal testing. Furthermore, they face challenges in evaluating complex substances like glyphosate in food matrices, where correlating clinical symptoms to specific exposure sources proves difficult^41^.

Network toxicology offers a contemporary solution to these limitations by harnessing bioinformatics and big data to map complex interactions between toxins and biological systems. This approach extends beyond traditional single-target analyses, examining multiple targets and their interconnected pathways to provide a more holistic understanding of glyphosate’s biological impacts^42^. Utilizing the ADMET database and network toxicology, the study predicted glyphosate’s potential for kidney toxicity and carcinogenicity. These predictions align with existing toxicity data, indicating that higher doses of glyphosate could increase the risk of adverse health effects, such as kidney injury and cancer.

Our identification of MMP9, MMP2, MMP8, MMP3, and PLG as critical hub proteins strongly suggests that glyphosate may perturb ECM homeostasis in renal tissues. This aligns with accumulating evidence linking ECM dysregulation to both acute kidney injury and the progression of renal fibrosis and cance^43^. Specifically, the consistent enrichment of “extracellular matrix disassembly” and “proteolysis” in GO analyses supports a model where glyphosate could promote tissue degradation and facilitate tumor invasion and metastasis. This finding complements previous studies reporting glyphosate-induced fibrotic changes in renal tissues^44^ but provides a more specific molecular underpinning by pinpointing MMPs and PLG as potential mediators. Furthermore, the high binding affinities predicted by molecular docking (ranging from − 5.03 to -6.29 kcal/mol) and stable binding observed in MD simulations for glyphosate complexed with MMP9, MMP8, MMP3, and PLG offer structural plausibility to this hypothesis. While MMPs are known targets in cancer biology^45^their specific implication in glyphosate-induced renal toxicity represents a significant mechanistic advancement elucidated by our network-based approach.

The KEGG pathway analysis revealed a prominent role for nitrogen metabolism in both glyphosate-induced kidney injury and cancer, exhibiting the highest enrichment for kidney cancer. This finding suggests a previously underappreciated mechanism of glyphosate toxicity. Glyphosate’s structural analogy to glycine, a key metabolite in nitrogen handling, raises the possibility that it could interfere with enzymatic processes involved in nitrogen balance, such as those catalyzed by carbonic anhydrases (CA2, CA9, CA12–identified as hub genes in the nitrogen metabolism pathway). Dysregulated nitrogen metabolism can lead to ammonia accumulation and oxidative stress^46^known contributors to renal damage and carcinogenesis. This potential metabolic disruption offers a new perspective on glyphosate toxicity, connecting its chemical structure to specific metabolic vulnerabilities in the kidney. While oxidative stress is a well-established consequence of glyphosate exposure^47^our data suggest nitrogen metabolism dysregulation might be an upstream or parallel event contributing significantly to renal pathology, warranting focused experimental investigation.

Our computational predictions align with and expand upon epidemiological and experimental evidence linking glyphosate to renal disease^48^. The predicted kidney injury and carcinogenicity risks from ADMET databases corroborate clinical concerns. The identification of common targets and pathways (e.g., ECM disassembly, bladder cancer pathway) between kidney injury and cancer in our analysis provides a plausible molecular explanation for epidemiological observations suggesting glyphosate exposure may increase the risk of chronic kidney disease and renal cell carcinoma^49^. It supports the concept that sustained injury and inflammation driven by glyphosate could create a microenvironment conducive to malignant transformation. However, our study is the first, to our knowledge, to computationally model these connections systematically using a network toxicology framework, highlighting shared hub proteins like MMPs and specific pathways like nitrogen metabolism as potential bridges between acute toxicity and chronic carcinogenesis.

While our integrated computational approach generated compelling hypotheses, several limitations must be acknowledged. Primarily, these predictions require rigorous experimental validation. In vitro studies are needed to confirm the binding of glyphosate to identified targets (MMP9, MMP2, MMP8, MMP3, PLG) and assess functional consequences, such as alterations in enzyme activity or ECM degradation rates. In vivo models of chronic, low-dose glyphosate exposure should be employed to measure changes in the expression and activity of these proteins within renal tissue, alongside markers of nitrogen metabolism and histological evaluation of kidney damage and pre-neoplastic changes. Secondly, while network toxicology excels at identifying potential interactions, it cannot definitively prove causality in a biological system. Future work should utilize techniques like RNA interference or targeted inhibitors against the identified hub proteins (e.g., MMP inhibitors) in glyphosate-exposed renal cells or animal models to assess their functional necessity in mediating toxicity. Thirdly, the potential contribution of glyphosate’s metabolite, AMPA, which also exhibits toxicity and environmental persistence [5], was not explicitly modeled here and warrants separate investigation. Finally, exploring potential interactions between the identified pathways (e.g., how ECM disruption influences nitrogen metabolism or vice versa) represents a crucial next step in understanding the systems-level impact of glyphosate.

## Conclusions

In summary, this study employed a robust computational strategy to propose that glyphosate may exert nephrotoxic and carcinogenic effects primarily through disrupting extracellular matrix integrity via interactions with MMPs and PLG, and by dysregulating nitrogen metabolism. These findings significantly advance our mechanistic understanding beyond general oxidative stress, offering specific, testable hypotheses for future research. The predicted interactions and identified pathways, particularly the novel link to nitrogen metabolism dysregulation, provide crucial focal points for designing targeted experimental studies to validate these mechanisms and assess their contribution to glyphosate-associated renal pathologies in biological systems. Ultimately, confirming these pathways could inform the development of preventive strategies or therapeutic interventions to mitigate the renal health risks associated with this ubiquitous herbicide.

## Data Availability

All data supporting the findings of this study are available within the paper and its Supplementary Information.

## References

[1] Gan, L. et al. Biomimetic photodegradation of glyphosate in Carborane-Functionalized nanoconfined spaces. *J. Am. Chem. Soc.* **145**, 13730–13741 (2023). 37338458 10.1021/jacs.3c02019 PMC10311523

[2] Masotti, F., Garavaglia, B. S., Gottig, N. & Ottado, J. Bioremediation of the herbicide glyphosate in polluted soils by plant-associated microbes. *Curr. Opin. Microbiol.* **73**, 102290 (2023). 36893683 10.1016/j.mib.2023.102290

[3] Hertel, R., Gibhardt, J., Martienssen, M., Kuhn, R. & Commichau, F. M. Molecular mechanisms underlying glyphosate resistance in bacteria. *Environ. Microbiol.* **23**, 2891–2905 (2021). 33876549 10.1111/1462-2920.15534

[4] Vicini, J. L., Reeves, W. R., Swarthout, J. T. & Karberg, K. A. Glyphosate in livestock: feed residues and animal health1. *J. Anim. Sci.* **97**, 4509–4518 (2019). 31495885 10.1093/jas/skz295 PMC6827263

[5] Van Bruggen, A. H. C. et al. Environmental and health effects of the herbicide glyphosate. *Sci. Total Environ.* **616–617**, 255–268 (2018). 29117584 10.1016/j.scitotenv.2017.10.309

[6] Wang, X. et al. Oxidative stress and metabolism: A mechanistic insight for glyphosate toxicology. *Annu. Rev. Pharmacol. Toxicol.* **62**, 617–639 (2022). 34990202 10.1146/annurev-pharmtox-020821-111552

[7] Marino, M. et al. Pleiotropic outcomes of glyphosate exposure: from organ damage to effects on inflammation, cancer, reproduction and development. *Int J. Mol. Sci* **22** (2021). 10.3390/ijms222212606 PMC8618927 34830483

[8] Lu, J. et al. Characterization of glyphosate-induced cardiovascular toxicity and apoptosis in zebrafish. *Sci. Total Environ.* **851**, 158308 (2022). 36030873 10.1016/j.scitotenv.2022.158308

[9] Li, W. et al. Association of glyphosate exposure with multiple adverse outcomes and potential mediators. *Chemosphere* **345**, 140477 (2023). 37858770 10.1016/j.chemosphere.2023.140477

[10] Anderson, G. Amyotrophic lateral sclerosis pathoetiology and pathophysiology: roles of astrocytes, gut microbiome, and muscle interactions via the mitochondrial melatonergic pathway, with disruption by Glyphosate-Based herbicides. *Int J. Mol. Sci* **24** (2022). 10.3390/ijms24010587 PMC9820185 36614029

[11] Martínez, M. A. et al. Use of human neuroblastoma SH-SY5Y cells to evaluate glyphosate-induced effects on oxidative stress, neuronal development and cell death signaling pathways. *Environ. Int.* **135**, 105414 (2020). 31874349 10.1016/j.envint.2019.105414

[12] Zhao, Z. et al. An integrated strategy combining network toxicology and feature-based molecular networking for exploring hepatotoxic constituents and mechanism of epimedii Folium-induced hepatotoxicity in vitro. *Food Chem. Toxicol.* **176**, 113785 (2023). 37080529 10.1016/j.fct.2023.113785

[13] Lin, Z., Basili, D. & Chou, W. C. Preface to the special issue of food and chemical toxicology on new approach methodologies and machine learning in food safety and chemical risk assessment: development of reproducible, open-source, and user-friendly tools for exposure, toxicokinetic, and toxicity assessments in the 21st century. *Food Chem. Toxicol.* **190**, 114809 (2024). 38857761 10.1016/j.fct.2024.114809

[14] Bhatt, P. et al. Binding interaction of glyphosate with glyphosate oxidoreductase and C-P lyase: molecular Docking and molecular dynamics simulation studies. *J. Hazard. Mater.* **409**, 124927 (2021). 33450511 10.1016/j.jhazmat.2020.124927

[15] Bhatt, P. et al. Bioremediation potential of laccase for catalysis of glyphosate, isoproturon, lignin, and parathion: molecular docking, dynamics, and simulation. *J. Hazard. Mater.* **443**, 130319 (2023). 36356521 10.1016/j.jhazmat.2022.130319

[16] Li, J. & Bi, H. Clarification of the molecular mechanisms underlying glyphosate-induced major depressive disorder: a network toxicology approach. *Ann. Gen. Psychiatry*. **23**, 8 (2024). 38297317 10.1186/s12991-024-00491-4 PMC10829247

[17] Lu, J. et al. Developmental toxicity and estrogenicity of glyphosate in zebrafish in vivo and in Silico studies. *Chemosphere* **343**, 140275 (2023). 37758082 10.1016/j.chemosphere.2023.140275

[18] He, J., Zhu, X., Xu, K., Li, Y. & Zhou, J. Network toxicological and molecular Docking to investigate the mechanisms of toxicity of agricultural chemical thiabendazole. *Chemosphere* **363**, 142711 (2024). 38964723 10.1016/j.chemosphere.2024.142711

[19] Chen, D. & Hou, X. Aspartame carcinogenic potential revealed through network toxicology and molecular Docking insights. *Sci. Rep.* **14**, 11492 (2024). 38769413 10.1038/s41598-024-62461-w PMC11106323

[20] He, Z. et al. Asiaticoside exerts neuroprotection through targeting NLRP3 inflammasome activation. *Phytomedicine* **127**, 155494 (2024). 38471370 10.1016/j.phymed.2024.155494

[21] Chen, S., Li, B., Chen, L. & Jiang, H. Uncovering the mechanism of Resveratrol in the treatment of diabetic kidney disease based on network pharmacology, molecular docking, and experimental validation. *J. Transl Med.* **21**, 380 (2023). 37308949 10.1186/s12967-023-04233-0 PMC10258995

[22] Xu, M. et al. Identification and validation of immune and oxidative stress-related diagnostic markers for diabetic nephropathy by WGCNA and machine learning. *Front. Immunol.* **14**, 1084531 (2023). 36911691 10.3389/fimmu.2023.1084531 PMC9992203

[23] Landrum, M. J. et al. ClinVar: public archive of interpretations of clinically relevant variants. *Nucleic Acids Res.* **44**, D862–868 (2016). 26582918 10.1093/nar/gkv1222 PMC4702865

[24] Zhu, M. et al. Exploring the mechanism of aloe-emodin in the treatment of liver cancer through network Pharmacology and cell experiments. *Front. Pharmacol.* **14**, 1238841 (2023). 37900162 10.3389/fphar.2023.1238841 PMC10600456

[25] Szklarczyk, D. et al. The STRING database in 2023: protein-protein association networks and functional enrichment analyses for any sequenced genome of interest. *Nucleic Acids Res.* **51**, D638–d646 (2023). 36370105 10.1093/nar/gkac1000 PMC9825434

[26] Aşır, A. & Aldudak, B. & Matur okur, N. The impact of postoperative albumin levels on Furosemide efficacy in infants with congenital heart disease. *Life (Basel)* 14 (2024). 10.3390/life14121679 PMC11727876 39768386

[27] Li, Y. et al. Air pollution and prostate cancer: unraveling the connection through network toxicology and machine learning. *Ecotoxicol. Environ. Saf.* **292**, 117966 (2025). 40022828 10.1016/j.ecoenv.2025.117966

[28] Song, H., Zhou, H., Yang, S. & He, C. Combining Mendelian randomization analysis and network toxicology strategy to identify causality and underlying mechanisms of environmental pollutants with glioblastoma: A study of Methyl-4-hydroxybenzoate. *Ecotoxicol. Environ. Saf.* **287**, 117311 (2024). 39536568 10.1016/j.ecoenv.2024.117311

[29] Kanehisa, M., Furumichi, M., Sato, Y., Matsuura, Y. & Ishiguro-Watanabe, M. KEGG: biological systems database as a model of the real world. *Nucleic Acids Res.* **53**, D672–d677 (2025). 39417505 10.1093/nar/gkae909 PMC11701520

[30] Kanehisa, M. Toward Understanding the origin and evolution of cellular organisms. *Protein Sci.* **28**, 1947–1951 (2019). 31441146 10.1002/pro.3715 PMC6798127

[31] Kanehisa, M. & Goto, S. KEGG: Kyoto encyclopedia of genes and genomes. *Nucleic Acids Res.* **28**, 27–30 (2000). 10592173 10.1093/nar/28.1.27 PMC102409

[32] Lan, Y., Peng, Q., Fu, B. & Liu, H. Effective analysis of thyroid toxicity and mechanisms of acetyltributyl citrate using network toxicology, molecular docking, and machine learning strategies. *Toxicology* **511**, 154029 (2025). 39657862 10.1016/j.tox.2024.154029

[33] Chen, L. et al. Molecular mechanism of oroxyli semen against triple-negative breast cancer verified by bioinformatics and in vitro experiments. *Med. (Baltim).* **102**, e34835 (2023). 10.1097/MD.0000000000034835 PMC10508518 37713894

[34] Guo, F. et al. Investigation of Pharmacological mechanism of natural product using pathway fingerprints similarity based on drug-target-pathway heterogenous network. *J. Cheminform*. **13**, 68 (2021). 34544480 10.1186/s13321-021-00549-5 PMC8454151

[35] Seeliger, D. & de Groot, B. L. Ligand Docking and binding site analysis with PyMOL and autodock/vina. *J. Comput. Aided Mol. Des.* **24**, 417–422 (2010). 20401516 10.1007/s10822-010-9352-6 PMC2881210

[36] Jo, S., Kim, T., Iyer, V. G. & Im, W. CHARMM-GUI: a web-based graphical user interface for CHARMM. *J. Comput. Chem.* **29**, 1859–1865 (2008). 18351591 10.1002/jcc.20945

[37] Mehmood, A., Nawab, S., Jia, G., Kaushik, A. C. & Wei, D. Q. Supervised screening of Tecovirimat-like compounds as potential inhibitors for the Monkeypox virus E8L protein. *J. Biomol. Struct. Dyn.* **42**, 8100–8113 (2024). 37561169 10.1080/07391102.2023.2245042

[38] Mehmood, A., Kaushik, A. C. & Wei, D. Q. Prediction and validation of potent peptides against herpes simplex virus type 1 via immunoinformatic and systems biology approach. *Chem. Biol. Drug Des.* **94**, 1868–1883 (2019). 31437863 10.1111/cbdd.13602

[39] Mehmood, A., Kaushik, A. C., Wang, Q., Li, C. D. & Wei, D. Q. Bringing structural implications and deep Learning-Based drug identification for KRAS mutants. *J. Chem. Inf. Model.* **61**, 571–586 (2021). 33513018 10.1021/acs.jcim.0c00488

[40] Mehmood, A., Li, D., Li, J., Kaushik, A. C. & Wei, D. Q. Supervised screening of EGFR inhibitors validated through computational structural biology approaches. *ACS Med. Chem. Lett.* **15**, 2190–2200 (2024). 39691517 10.1021/acsmedchemlett.4c00385 PMC11647682

[41] Lauterstein, D., Savidge, M., Chen, Y., Weil, R. & Yeager, R. P. Nonanimal toxicology testing approaches for traditional and deemed tobacco products in a complex regulatory environment: limitations, possibilities, and future directions. *Toxicol. Vitro*. **62**, 104684 (2020). 10.1016/j.tiv.2019.104684 31618670

[42] Zhang, D. et al. Network Pharmacology modeling identifies synergistic interaction of therapeutic and toxicological mechanisms for tripterygium hypoglaucum Hutch. *BMC Complement. Med. Ther.* **21**, 38 (2021). 33446184 10.1186/s12906-021-03210-8 PMC7809745

[43] Li, L., Fu, H. & Liu, Y. The fibrogenic niche in kidney fibrosis: components and mechanisms. *Nat. Rev. Nephrol.* **18**, 545–557 (2022). 35788561 10.1038/s41581-022-00590-z

[44] Ding, F. et al. Melatonin ameliorates renal dysfunction in glyphosate- and hard water-treated mice. *Ecotoxicol. Environ. Saf.* **241**, 113803 (2022). 36068739 10.1016/j.ecoenv.2022.113803

[45] Cox, T. R. The matrix in cancer. *Nat. Rev. Cancer*. **21**, 217–238 (2021). 33589810 10.1038/s41568-020-00329-7

[46] Skowrońska, M. & Albrecht, J. Oxidative and nitrosative stress in ammonia neurotoxicity. *Neurochem Int.* **62**, 731–737 (2013). 23142151 10.1016/j.neuint.2012.10.013

[47] Chang, V. C. et al. Glyphosate exposure and urinary oxidative stress biomarkers in the agricultural health study. *J. Natl. Cancer Inst.* **115**, 394–404 (2023). 36629488 10.1093/jnci/djac242 PMC10086635

[48] Hu, L., Chen, M., Xue, X., Zhao, M. & He, Q. Effect of glyphosate on renal function: A study integrating epidemiological and experimental evidence. *Ecotoxicol. Environ. Saf.* **290**, 117758 (2025). 39862699 10.1016/j.ecoenv.2025.117758

[49] Soerensen, S. J. C. et al. Groundwater constituents and the incidence of kidney cancer. *Cancer* **129**, 3309–3317 (2023). 37287332 10.1002/cncr.34898

